# Supplementary material for: AI-Based Risk Score from Tumour-Infiltrating Lymphocyte Predicts Locoregional-Free Survival in Nasopharyngeal Carcinoma
Source: Cancers (Basel). 2023 Dec 10;15(24):5789. doi: 10.3390/cancers15245789 (PMC10742296; doi:10.3390/cancers15245789)
Supplement: Supplementary file 1 [file cancers-15-05789-s001.zip › cancers-2716479-supplementary.pdf]

Figure S1 Nuclei Classification Model

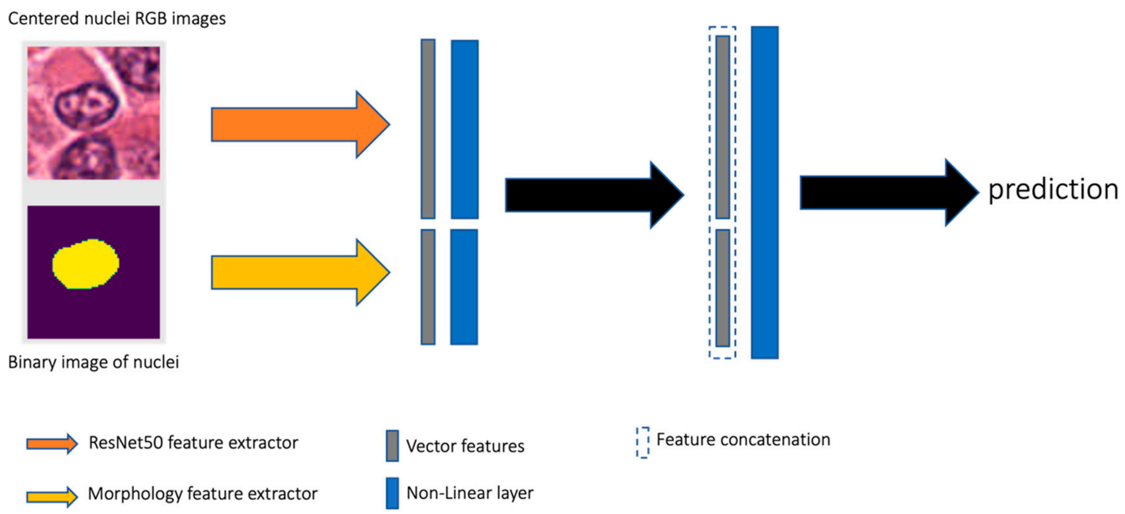

Figure S2 Ranking of Feature Importance

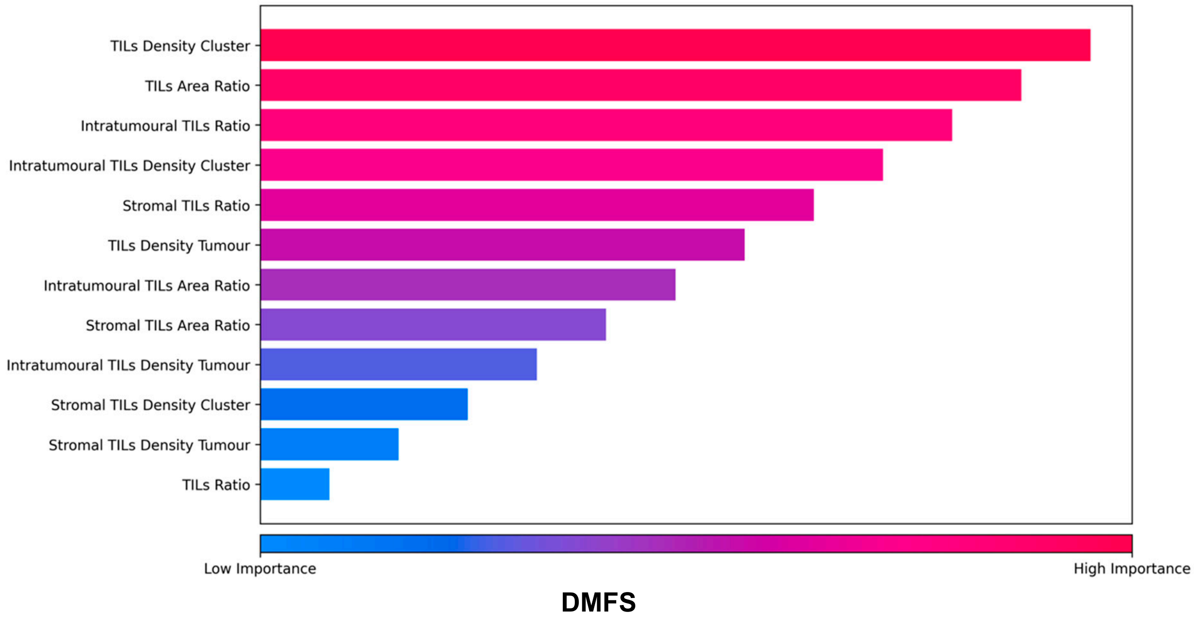

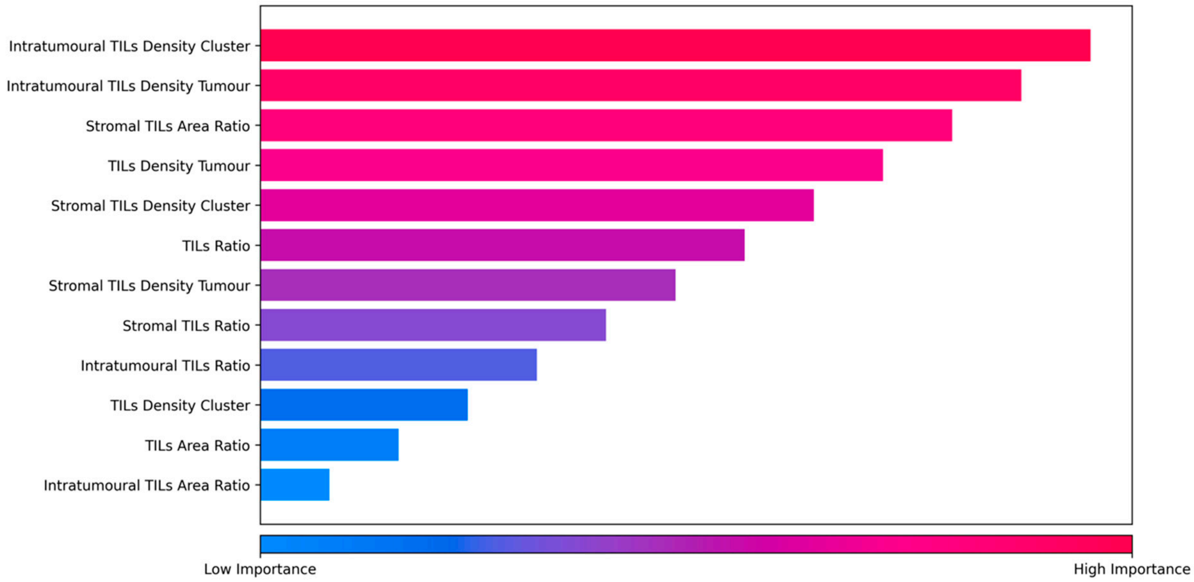
Intratumoural TILs Density Cluster
Intratumoural TILs Density Tumour
Stromal TILs Area Ratio
TILs Density Tumour
Stromal TILs Density Cluster
TILs Ratio
Stromal TILs Density Tumour
Stromal TILs Ratio
Intratumoural TILs Ratio
TILs Density Cluster
TILs Area Ratio
Intratumoural TILs Area Ratio
Low Importance
High Importance

PFS

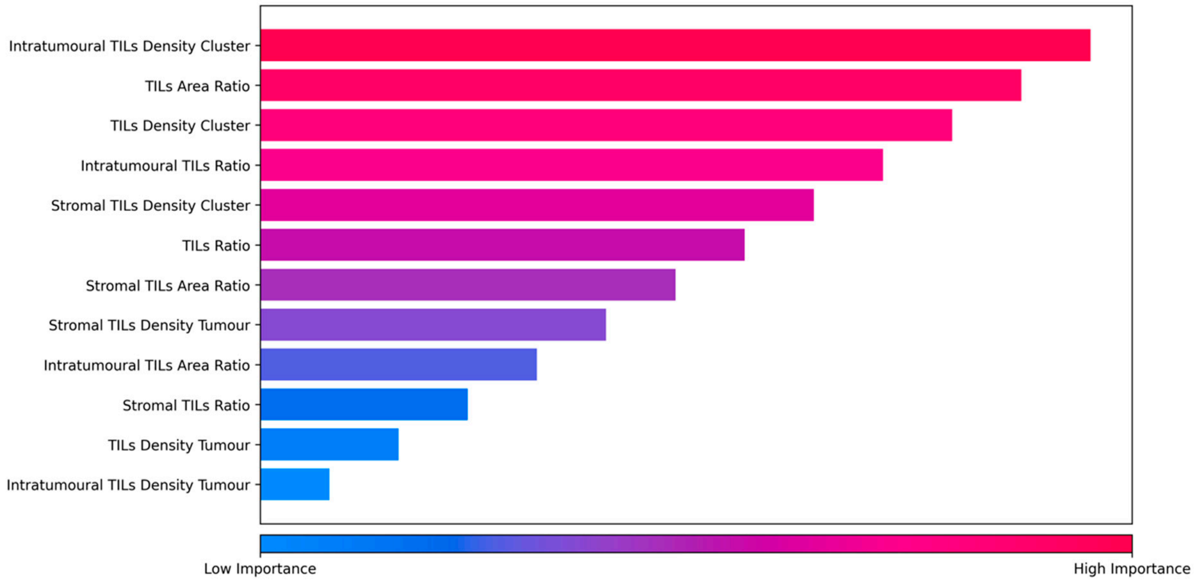
Intratumoural TILs Density Cluster
TILs Area Ratio
TILs Density Cluster
Intratumoural TILs Ratio
Stromal TILs Density Cluster
TILs Ratio
Stromal TILs Area Ratio
Stromal TILs Density Tumour
Intratumoural TILs Area Ratio
Stromal TILs Ratio
TILs Density Tumour
Intratumoural TILs Density Tumour
Low Importance
High Importance

RRFS

Table S1 Digital NPC-TILs Formula

NO	FEATURE NAMES	FORMULA	DESCRIPTION
1	TILs Ratio	$\frac{\sum_{x=1}^{n}\frac{(K_{ix}+K_{ox})}{(J_{ix}+J_{ox})}}{n}$	The ratio between number of lymphocytes cells with tumour cells inside and around the tumour clusters
2	Intratumoural TILs Ratio	$\frac{\sum_{x=1}^{n}\frac{K_{ix}}{J_{ix}}}{n}$	Ratio between number lymphocytes cells with tumour cells inside the tumour cluster
3	Stromal TILs Ratio	$\frac{\sum_{x=1}^{n}\frac{K_{ox}}{J_{ox}}}{n}$	Ratio between number lymphocytes cells with tumour cells in the periphery of tumour cluster
4	TILs Density Cluster	$\frac{\sum_{x=1}^{n}\frac{(K_{ix}+K_{ox})}{(C_{ix}+C_{ox})}}{n}$	Number of lymphocyte cells within the tumour cluster area and the expanded area of the tumour cluster
5	Intratumoural TILs Density Cluster	$\frac{\sum_{x=1}^{n}\frac{K_{ix}}{C_{ix}}}{n}$	Number of lymphocyte cells within the tumour cluster area only
6	Stromal TILs Density Cluster	$\frac{\sum_{x=1}^{n}\frac{K_{ox}}{C_{ox}}}{n}$	Number of lymphocyte cells within the expanded area of the tumour cluster only
7	TILs Density Tumour	$\frac{\sum_{x=1}^{n}\frac{(K_{ix}+K_{ox})}{(B_{ix}+B_{ox})}}{n}$	Proportion between number of lymphocyte cells with area of tumour cells inside and around the tumour clusters
8	Intratumoural TILs Density Tumour	$\frac{\sum_{x=1}^{n}\frac{K_{ix}}{B_{ix}}}{n}$	Proportion between number of lymphocyte cells with area of tumour cells inside the tumour clusters
9	Stromal TILs Density Tumour	$\frac{\sum_{x=1}^{n}\frac{K_{ox}}{B_{ox}}}{n}$	Proportion between number of lymphocyte cells with area of tumour cells in the periphery of tumour clusters
10	TILs Area Ratio	$\frac{\sum_{x=1}^{n}\frac{(A_{ix}+A_{ox})}{(B_{ix}+B_{ox})}}{n}$	Ratio of lymphocytes cells area and tumour cells area inside and around the tumour clusters
11	Intratumoural TILs Area Ratio	$\frac{\sum_{x=1}^{n}\frac{A_{ix}}{B_{ix}}}{n}$	Ratio of lymphocytes cells area and tumour cells area inside the tumour clusters

NO	FEATURE NAMES	FORMULA	DESCRIPTION
12	Stromal TILs Area Ratio	$\frac{\sum_{x=1}^{n} \frac{A_{ox}}{B_{ox}}}{n}$	Ratio of lymphocytes cells area and tumour cells area in the periphery of tumour clusters

Description:

- n is the total number of clusters in slide
- i is inner tumour cluster
- o is outer tumour cluster
- K is the total number of lymphocyte cells in cluster
- J is the total number of tumour cells in cluster
- A is the total area of lymphocyte cells in cluster
- B is the total area of tumour cells in cluster
- C is the area of cluster

Table S2 Three-Fold Cross-Validation Result of Concordance Index for RSF Model

ENDPOINTS	FEATURES	C-Index (mean-std) DISCOVERY	C-Index (mean-std) VALIDATION
DMFS	Clinical	0.764 ± 0.009	**0.670 ± 0.040**
	Digital NPC-TILs	0.835 ± 0.003	0.645 ± 0.031
	TM	0.839 ± 0.010	0.566 ± 0.013
	Digital NPC-TILs & Clinical	0.840 ± 0.016	0.628 ± 0.080
	Digital TILs & TM	0.822 ± 0.018	0.623 ± 0.033
	Clinical & TM	0.851 ± 0.022	0.660 ± 0.032
	All features	0.862 ± 0.028	0.658 ± 0.072
RRFS	Clinical	0.937 ± 0.019	0.775 ± 0.020
	Digital TILs	0.972 ± 0.016	**0.780 ± 0.030**
	TM	0.960 ± 0.023	0.713 ± 0.093
	Digital TILs & Clinical	0.990 ± 0.007	0.737 ± 0.107
	Digital TILs & TM	0.974 ± 0.026	0.724 ± 0.132
	Clinical & TM	0.977 ± 0.014	0.754 ± 0.107
	All features	0.988 ± 0.010	0.652 ± 0.139
PFS	Clinical	0.754 ± 0.021	0.583 ± 0.016
	Digital TILs	0.789 ± 0.027	**0.667 ± 0.026**
	TM	0.819 ± 0.008	0.574 ± 0.043
	Digital TILs & Clinical	0.846 ± 0.015	0.589 ± 0.080
	Digital TILs & TM	0.817 ± 0.007	0.579 ± 0.027
	Clinical & TM	0.825 ± 0.007	0.632 ± 0.030
	All features	0.834 ± 0.016	0.587 ± 0.045

Table S3 Univariate Analysis on DMFS

Covariate	Sub-covariate	HR	Lower HR 95%	Upper HR 95%	p-values
Age		0.96	0.91	1	0.0554
Gender	Female	references			
	Male	0.5	0.18	1.41	0.193
T	1	references			
	2	0.72	0.08	6.33	0.7704
	3	0.52	0.08	3.35	0.4935
	4	0.53	0.07	3.84	0.5274
N	0	References			
	1	0.58	0.10	3.53	0.5546
	2	2.08	0.42	10.36	0.3721
	3	3.18	0.60	16.74	0.1726
Stage	I	References			
	II	0.07	0.00	5.2	0.2292
	III	0.34	0.04	2.74	0.3118
	IV	0.56	0.07	4.51	0.5889
EBV DNA copies	≤ 4000	References			
	> 4000	3.88	1.21	12.47	**<0.05**
Digital TILs		1.36	1.14	1.61	**<0.001**

Table S4 Univariate Analysis on RRFS

Covariate	Sub-covariate	HR	Lower HR 95%	Upper HR 95%	p-values
Age		1.03	0.94	1.14	0.5395
Gender	Female	references			
	Male	0.60	0.06	5.91	0.6639
T	1	references			
	2	0.50	0.00	54.24	0.7696
	3	1.18	0.08	17.21	0.9015
	4	2.34	0.14	39.83	0.5567
N	0	references			
	1	0.44	0.02	9.11	0.5955
	2	2.03	0.13	30.93	0.6117
	3	4.18	0.22	80.03	0.3425
Stage	I	references			
	II	0.46	0.00	67.87	0.7587
	III	1.01	0.06	16.93	0.9946

Covariate	Sub-covariate	HR	Lower HR 95%	Upper HR 95%	p-values
	IV	1.39	0.08	24.38	0.8198
EBV DNA copies	≤ 4000	references			
	> 4000	4.60	0.37	56.48	0.2329
Risk Scores		1.65	0.85	3.21	0.1388

Table S5 Univariate Analysis on PFS

Covariate	Sub-covariate	HR	Lower HR 95%	Upper HR 95%	p-values
Age		1	0.96	1.04	0.9953
Gender	Female	references			
	Male	0.91	0.35	2.33	0.8393
T	1	references			
	2	0.81	0.08	7.89	0.8527
	3	1.09	0.23	5.26	0.9106
	4	2.76	0.57	13.32	0.2061
N	0	references			
	1	1.51	0.32	7.14	0.6032
	2	0.83	0.15	4.46	0.8277
	**3**	6.7	1.45	30.88	**<0.05**
Stage	1	references			
	2	0.19	0	18.83	0.4812
	3	1.18	0.14	9.79	0.8781
	4	5.21	0.65	41.55	0.1194
EBV DNA copies	≤ 4000	references			
	> 4000	1.14	0.47	2.74	0.7687
Risk Scores		1.08	1.03	1.14	**<0.005**

Table S6 Multivariate Analysis on DMFS

Covariate	Sub-covariate	HR	Lower HR 95%	Upper HR 95%	p-values
Age		0.97	0.92	1.03	0.3506
Gender	Female	references			
	Male	0.28	0.08	0.97	**<0.05**
T	1	references			
	2	1.73	0.13	22.93	0.6790
	3	0.47	0.05	4.05	0.4898
	4	0.63	0.05	7.23	0.7097
N	0	references			
	1	0.62	0.08	4.76	0.6456
	2	1.70	0.23	12.67	0.6028
	3	1.56	0.12	19.70	0.7239
Stage	I	references			
	II	0.09	0.00	7.72	0.2854
	III	0.64	0.06	7.26	0.7205
	IV	0.71	0.06	8.85	0.7916
EBV DNA copies	≤ 4000	references			
	> 4000	2.52	0.73	8.71	0.1450
Risk Scores		1.35	1.1	1.67	**<0.005**

Table S7 Multivariate Analysis on RRFS

Covariate	Sub-covariate	HR	Lower HR 95%	Upper HR 95%	p-values
Age		1.04	0.94	1.16	0.4175
Gender	Female	references			
	Male	0.41	0.03	5.31	0.4928
T	1	references			
	2	0.61	0.00	92.50	0.8466
	3	1.03	0.06	18.99	0.9847
	4	3.33	0.15	73.37	0.4454
N	0	references			
	1	0.33	0.02	6.59	0.4696
	2	1.40	0.07	27.88	0.8270
	3	6.72	0.23	194.73	0.2675
Stage	I	references			
	II	0.79	0.00	280.62	0.9363
	III	1.61	0.07	39.43	0.7688

Covariate	Sub-covariate	HR	Lower HR 95%	Upper HR 95%	p-values
	IV	0.65	0.02	17.04	0.7970
EBV DNA copies	≤ 4000	references			
	> 4000	5.74	0.45	73.55	0.1793
Risk Scores		2.03	0.91	4.49	0.0826

Table S8 Multivariate Analysis on PFS

Covariate	Sub-covariate	HR	Lower HR 95%	Upper HR 95%	p-values
Age		1.02	0.98	1.06	0.3655
Gender	Female	references			
	Male	0.79	0.28	2.25	0.6562
T	1	references			
	2	0.72	0.06	8.83	0.7938
	3	0.68	0.10	4.42	0.6860
	4	0.74	0.10	5.53	0.7651
N	0	references			
	1	1.00	0.18	5.57	0.9984
	2	0.74	0.12	4.55	0.7467
	3	3.15	0.46	21.39	0.2397
Stage	I	references			
	II	0.21	0.00	25.04	0.5237
	III	1.45	0.14	15.50	0.7593
	IV	3.44	0.30	38.89	0.3177
EBV DNA copies	≤ 4000	references			
	> 4000	1.11	0.43	2.89	0.8287
Risk Scores		1.08	1.02	1.14	**<0.05**

Table S9 Tumour Morphology Features

No	Feature	Description
1	Bounding Box	Rectangular box that encloses cell
2	Convex Area	Area of the smallest convex shape that contains all the contour points
3	Contour Area	Area enclosed by the boundary of cell based on its contour
4	Major Axis Length	Longest diameter of nuclei
5	Perimeter	Total length of cell boundary
6	Radius	Distance between center of nucleus to its boundary
7	Solidity	Proportion of the cell pixels that belong to the ROI compared to the total pixels in the convex hull of the cell
8	Equivalent Diameter	Diameter of a circle that has the same are as the cell
9	Eccentricity	Circularity of cell
